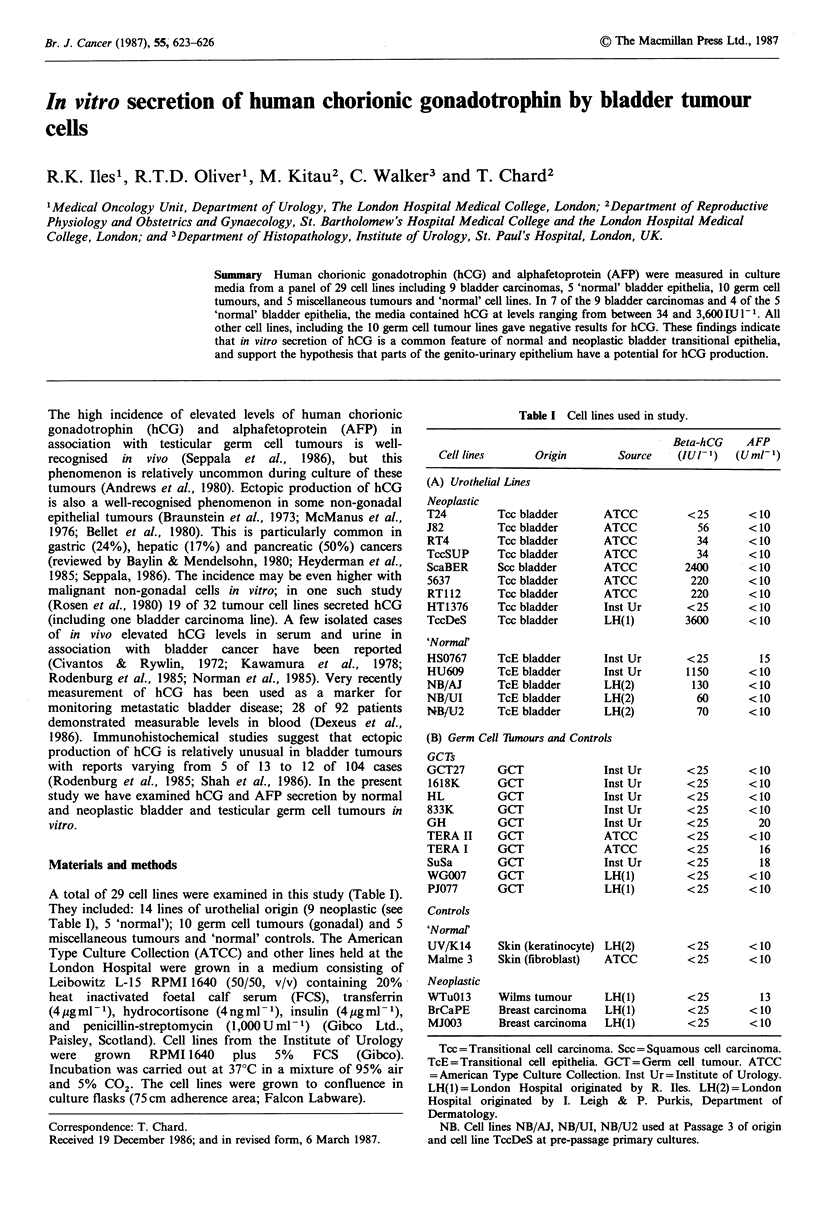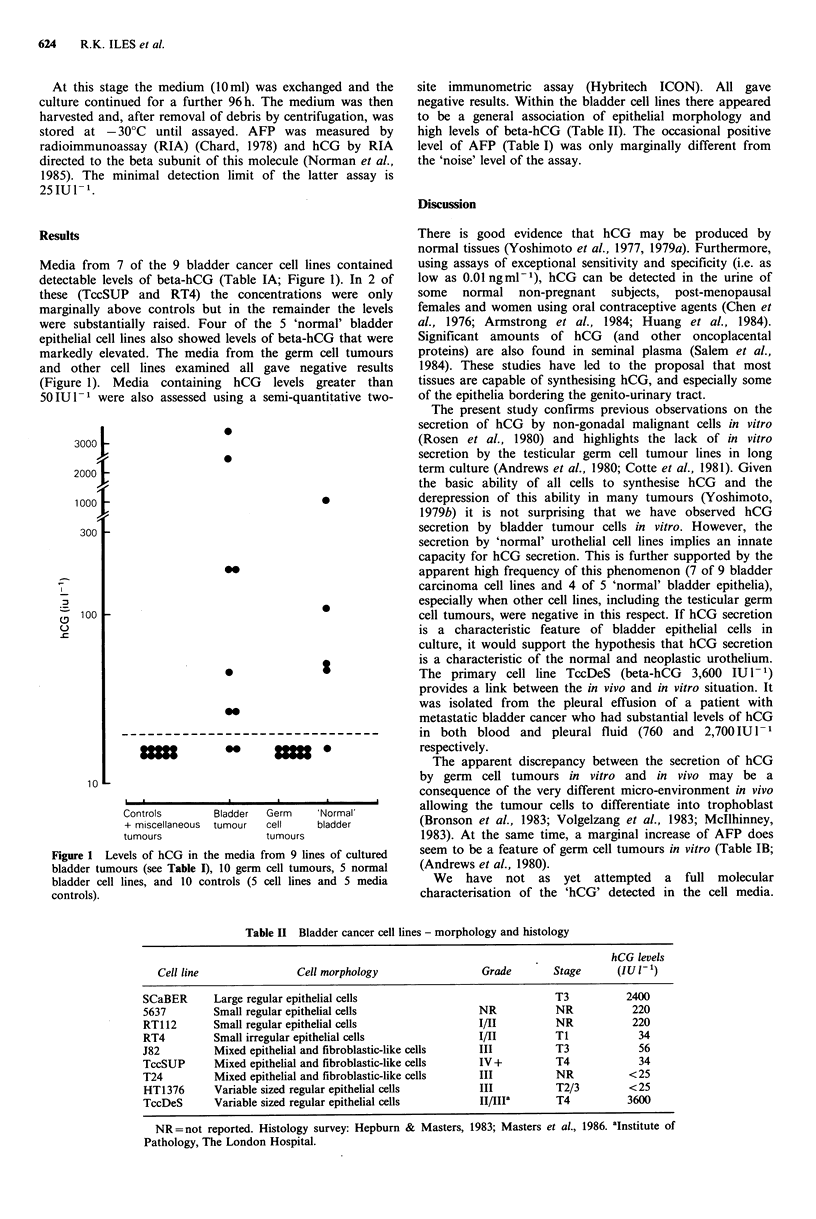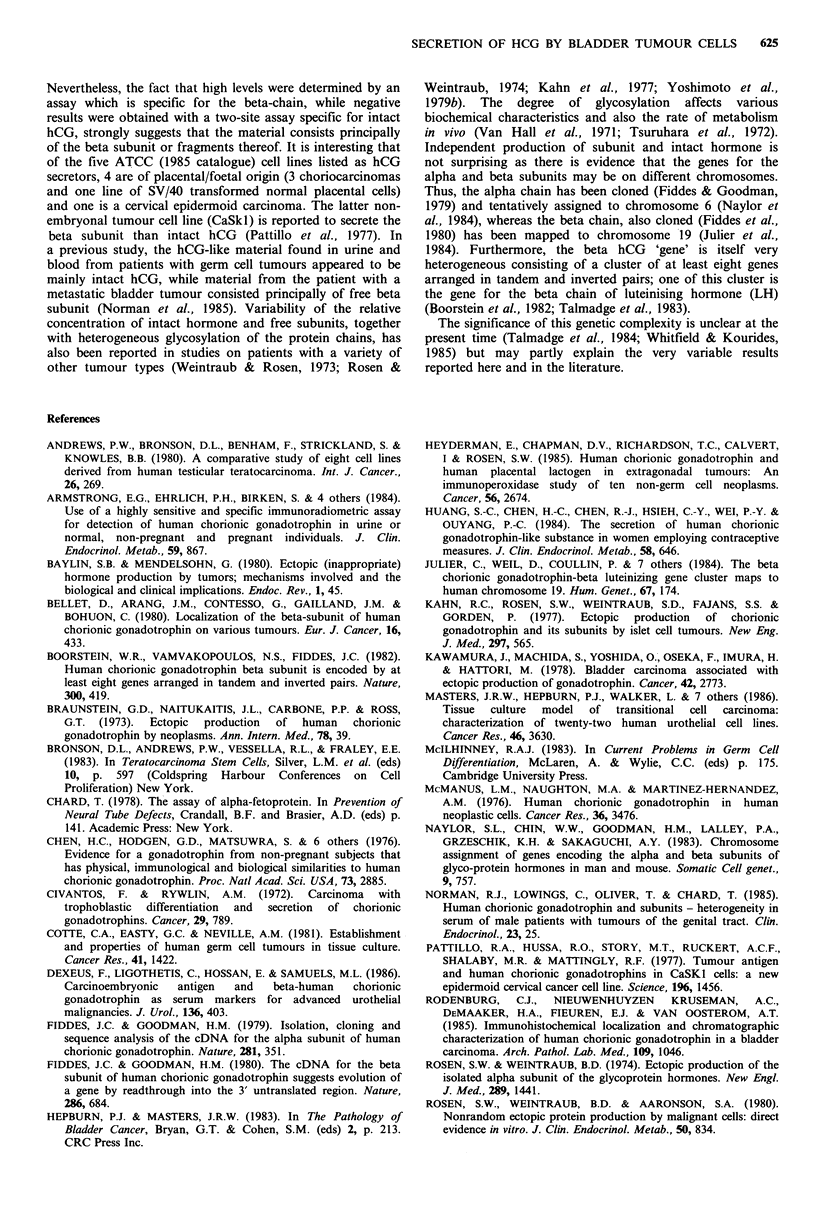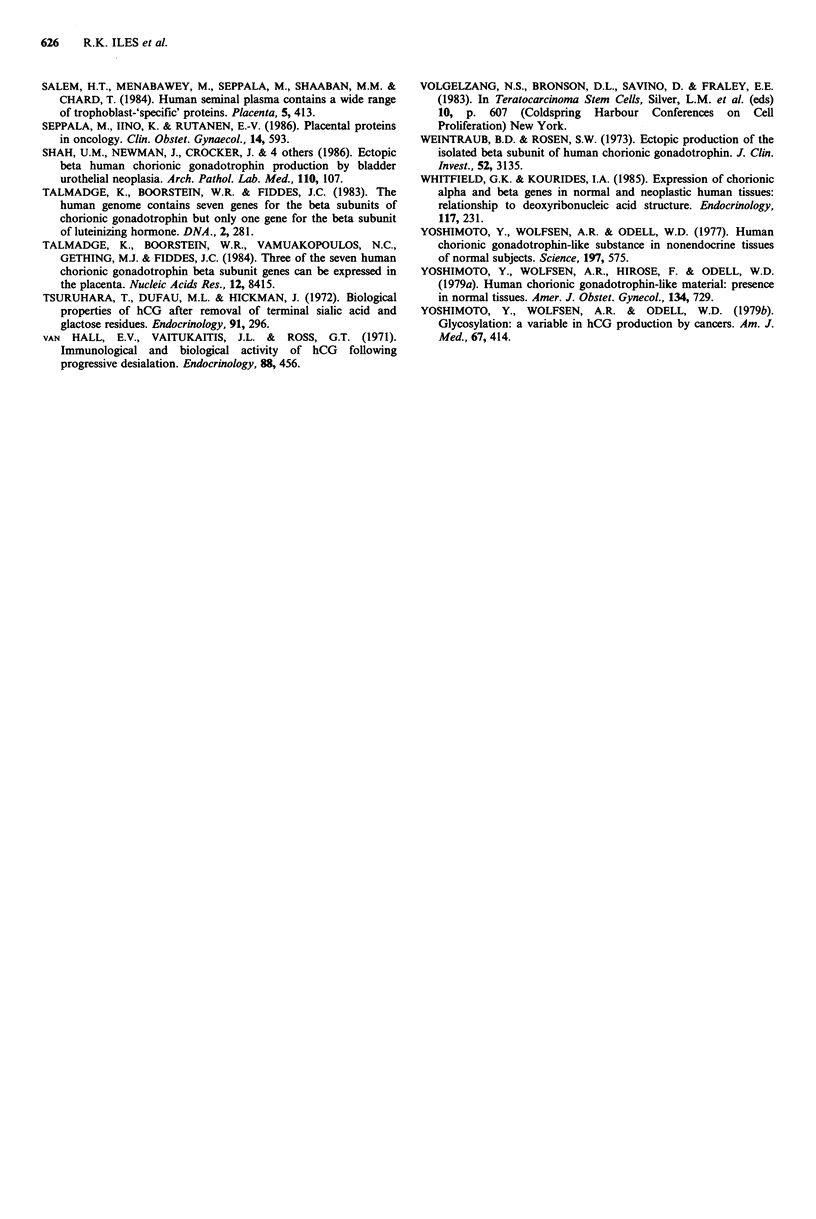# In vitro secretion of human chorionic gonadotrophin by bladder tumour cells.

**DOI:** 10.1038/bjc.1987.126

**Published:** 1987-06

**Authors:** R. K. Iles, R. T. Oliver, M. Kitau, C. Walker, T. Chard

## Abstract

Human chorionic gonadotrophin (hCG) and alphafetoprotein (AFP) were measured in culture media from a panel of 29 cell lines including 9 bladder carcinomas, 5 'normal' bladder epithelia, 10 germ cell tumours, and 5 miscellaneous tumours and 'normal' cell lines. In 7 of the 9 bladder carcinomas and 4 of the 5 'normal' bladder epithelia, the media contained hCG at levels ranging from between 34 and 3,600 IU l(-1). All other cell lines, including the 10 germ cell tumour lines gave negative results for hCG. These findings indicate that in vitro secretion of hCG is a common feature of normal and neoplastic bladder transitional epithelia, and support the hypothesis that parts of the genito-urinary epithelium have a potential for hCG production.


*Br. J. Cancer* (1987), **55**, 623–626 © The Macmillan Press Ltd., 1987

### *In vitro* secretion of human chorionic gonadotrophin by bladder tumour cells

R.K. Iles[1], R.T.D. Oliver[1], M. Kitau[2], C. Walker[3] and T. Chard[2]

[1]*Medical Oncology Unit, Department of Urology, The London Hospital Medical College, London;* [2]*Department of Reproductive Physiology and Obstetrics and Gynaecology, St. Bartholomew's Hospital Medical College and the London Hospital Medical College, London; and* [3]*Department of Histopathology, Institute of Urology, St. Paul's Hospital, London, UK.*

**Summary** Human chorionic gonadotrophin (hCG) and alphafetoprotein (AFP) were measured in culture media from a panel of 29 cell lines including 9 bladder carcinomas, 5 'normal' bladder epithelia, 10 germ cell tumours, and 5 miscellaneous tumours and 'normal' cell lines. In 7 of the 9 bladder carcinomas and 4 of the 5 'normal' bladder epithelia, the media contained hCG at levels ranging from between 34 and 3,600 IU $l^{-1}$. All other cell lines, including the 10 germ cell tumour lines gave negative results for hCG. These findings indicate that *in vitro* secretion of hCG is a common feature of normal and neoplastic bladder transitional epithelia, and support the hypothesis that parts of the genito-urinary epithelium have a potential for hCG production.

The high incidence of elevated levels of human chorionic gonadotrophin (hCG) and alphafetoprotein (AFP) in association with testicular germ cell tumours is well-recognised *in vivo* (Seppala *et al.*, 1986), but this phenomenon is relatively uncommon during culture of these tumours (Andrews *et al.*, 1980). Ectopic production of hCG is also a well-recognised phenomenon in some non-gonadal epithelial tumours (Braunstein *et al.*, 1973; McManus *et al.*, 1976; Bellet *et al.*, 1980). This is particularly common in gastric (24%), hepatic (17%) and pancreatic (50%) cancers (reviewed by Baylin & Mendelsohn, 1980; Heyderman *et al.*, 1985; Seppala, 1986). The incidence may be even higher with malignant non-gonadal cells *in vitro*; in one such study (Rosen *et al.*, 1980) 19 of 32 tumour cell lines secreted hCG (including one bladder carcinoma line). A few isolated cases of *in vivo* elevated hCG levels in serum and urine in association with bladder cancer have been reported (Civantos & Rywlin, 1972; Kawamura *et al.*, 1978; Rodenburg *et al.*, 1985; Norman *et al.*, 1985). Very recently measurement of hCG has been used as a marker for monitoring metastatic bladder disease; 28 of 92 patients demonstrated measurable levels in blood (Dexeus *et al.*, 1986). Immunohistochemical studies suggest that ectopic production of hCG is relatively unusual in bladder tumours with reports varying from 5 of 13 to 12 of 104 cases (Rodenburg *et al.*, 1985; Shah *et al.*, 1986). In the present study we have examined hCG and AFP secretion by normal and neoplastic bladder and testicular germ cell tumours *in vitro*.

### Materials and methods

A total of 29 cell lines were examined in this study (Table I). They included: 14 lines of urothelial origin (9 neoplastic (see Table I), 5 'normal'); 10 germ cell tumours (gonadal) and 5 miscellaneous tumours and 'normal' controls. The American Type Culture Collection (ATCC) and other lines held at the London Hospital were grown in a medium consisting of Leibowitz L-15 RPMI 1640 (50/50, v/v) containing 20% heat inactivated foetal calf serum (FCS), transferrin (4 $\mu$g $ml^{-1}$), hydrocortisone (4 ng $ml^{-1}$), insulin (4 $\mu$g $ml^{-1}$), and penicillin-streptomycin (1,000 U $ml^{-1}$) (Gibco Ltd., Paisley, Scotland). Cell lines from the Institute of Urology were grown RPMI 1640 plus 5% FCS (Gibco). Incubation was carried out at 37°C in a mixture of 95% air and 5% $CO_2$. The cell lines were grown to confluence in culture flasks (75 cm adherence area; Falcon Labware).

Correspondence: T. Chard.
Received 19 December 1986; and in revised form, 6 March 1987.

**Table I** Cell lines used in study.

| *Cell lines* | *Origin* | *Source* | *Beta-hCG* ($IU\,l^{-1}$) | *AFP* ($U\,ml^{-1}$) |
|---|---|---|---|---|
| (A) *Urothelial Lines* | | | | |
| *Neoplastic* | | | | |
| T24 | Tcc bladder | ATCC | <25 | <10 |
| J82 | Tcc bladder | ATCC | 56 | <10 |
| RT4 | Tcc bladder | ATCC | 34 | <10 |
| TccSUP | Tcc bladder | ATCC | 34 | <10 |
| ScaBER | Scc bladder | ATCC | 2400 | <10 |
| 5637 | Tcc bladder | ATCC | 220 | <10 |
| RT112 | Tcc bladder | ATCC | 220 | <10 |
| HT1376 | Tcc bladder | Inst Ur | <25 | <10 |
| TccDeS | Tcc bladder | LH(1) | 3600 | <10 |
| *'Normal'* | | | | |
| HS0767 | TcE bladder | Inst Ur | <25 | 15 |
| HU609 | TcE bladder | Inst Ur | 1150 | <10 |
| NB/AJ | TcE bladder | LH(2) | 130 | <10 |
| NB/UI | TcE bladder | LH(2) | 60 | <10 |
| NB/U2 | TcE bladder | LH(2) | 70 | <10 |
| (B) *Germ Cell Tumours and Controls* | | | | |
| *GCTs* | | | | |
| GCT27 | GCT | Inst Ur | <25 | <10 |
| 1618K | GCT | Inst Ur | <25 | <10 |
| HL | GCT | Inst Ur | <25 | <10 |
| 833K | GCT | Inst Ur | <25 | <10 |
| GH | GCT | Inst Ur | <25 | 20 |
| TERA II | GCT | ATCC | <25 | <10 |
| TERA I | GCT | ATCC | <25 | 16 |
| SuSa | GCT | Inst Ur | <25 | 18 |
| WG007 | GCT | LH(1) | <25 | <10 |
| PJ077 | GCT | LH(1) | <25 | <10 |
| *Controls* | | | | |
| *'Normal'* | | | | |
| UV/K14 | Skin (keratinocyte) | LH(2) | <25 | <10 |
| Malme 3 | Skin (fibroblast) | ATCC | <25 | <10 |
| *Neoplastic* | | | | |
| WTu013 | Wilms tumour | LH(1) | <25 | 13 |
| BrCaPE | Breast carcinoma | LH(1) | <25 | <10 |
| MJ003 | Breast carcinoma | LH(1) | <25 | <10 |

Tcc = Transitional cell carcinoma. Scc = Squamous cell carcinoma. TcE = Transitional cell epithelia. GCT = Germ cell tumour. ATCC = American Type Culture Collection. Inst Ur = Institute of Urology. LH(1) = London Hospital originated by R. Iles. LH(2) = London Hospital originated by I. Leigh & P. Purkis, Department of Dermatology.

NB. Cell lines NB/AJ, NB/UI, NB/U2 used at Passage 3 of origin and cell line TccDeS at pre-passage primary cultures.

At this stage the medium (10 ml) was exchanged and the culture continued for a further 96 h. The medium was then harvested and, after removal of debris by centrifugation, was stored at −30°C until assayed. AFP was measured by radioimmunoassay (RIA) (Chard, 1978) and hCG by RIA directed to the beta subunit of this molecule (Norman et al., 1985). The minimal detection limit of the latter assay is 25 IU $l^{-1}$.

### Results

Media from 7 of the 9 bladder cancer cell lines contained detectable levels of beta-hCG (Table IA; Figure 1). In 2 of these (TccSUP and RT4) the concentrations were only marginally above controls but in the remainder the levels were substantially raised. Four of the 5 'normal' bladder epithelial cell lines also showed levels of beta-hCG that were markedly elevated. The media from the germ cell tumours and other cell lines examined all gave negative results (Figure 1). Media containing hCG levels greater than 50 IU $l^{-1}$ were also assessed using a semi-quantitative two-site immunometric assay (Hybritech ICON). All gave negative results. Within the bladder cell lines there appeared to be a general association of epithelial morphology and high levels of beta-hCG (Table II). The occasional positive level of AFP (Table I) was only marginally different from the 'noise' level of the assay.

**Figure 1** Levels of hCG in the media from 9 lines of cultured bladder tumours (see **Table I**), 10 germ cell tumours, 5 normal bladder cell lines, and 10 controls (5 cell lines and 5 media controls).

### Discussion

There is good evidence that hCG may be produced by normal tissues (Yoshimoto et al., 1977, 1979a). Furthermore, using assays of exceptional sensitivity and specificity (i.e. as low as 0.01 ng $ml^{-1}$), hCG can be detected in the urine of some normal non-pregnant subjects, post-menopausal females and women using oral contraceptive agents (Chen et al., 1976; Armstrong et al., 1984; Huang et al., 1984). Significant amounts of hCG (and other oncoplacental proteins) are also found in seminal plasma (Salem et al., 1984). These studies have led to the proposal that most tissues are capable of synthesising hCG, and especially some of the epithelia bordering the genito-urinary tract.

The present study confirms previous observations on the secretion of hCG by non-gonadal malignant cells in vitro (Rosen et al., 1980) and highlights the lack of in vitro secretion by the testicular germ cell tumour lines in long term culture (Andrews et al., 1980; Cotte et al., 1981). Given the basic ability of all cells to synthesise hCG and the derepression of this ability in many tumours (Yoshimoto, 1979b) it is not surprising that we have observed hCG secretion by bladder tumour cells in vitro. However, the secretion by 'normal' urothelial cell lines implies an innate capacity for hCG secretion. This is further supported by the apparent high frequency of this phenomenon (7 of 9 bladder carcinoma cell lines and 4 of 5 'normal' bladder epithelia), especially when other cell lines, including the testicular germ cell tumours, were negative in this respect. If hCG secretion is a characteristic feature of bladder epithelial cells in culture, it would support the hypothesis that hCG secretion is a characteristic of the normal and neoplastic urothelium. The primary cell line TccDeS (beta-hCG 3,600 IU $l^{-1}$) provides a link between the in vivo and in vitro situation. It was isolated from the pleural effusion of a patient with metastatic bladder cancer who had substantial levels of hCG in both blood and pleural fluid (760 and 2,700 IU $l^{-1}$ respectively.

The apparent discrepancy between the secretion of hCG by germ cell tumours in vitro and in vivo may be a consequence of the very different micro-environment in vivo allowing the tumour cells to differentiate into trophoblast (Bronson et al., 1983; Volgelzang et al., 1983; McIlhinney, 1983). At the same time, a marginal increase of AFP does seem to be a feature of germ cell tumours in vitro (Table IB; (Andrews et al., 1980).

We have not as yet attempted a full molecular characterisation of the 'hCG' detected in the cell media.

**Table II** Bladder cancer cell lines – morphology and histology

| *Cell line* | *Cell morphology* | *Grade* | *Stage* | *hCG levels ($IU\,l^{-1}$)* |
|---|---|---|---|---|
| SCaBER | Large regular epithelial cells | | T3 | 2400 |
| 5637 | Small regular epithelial cells | NR | NR | 220 |
| RT112 | Small regular epithelial cells | I/II | NR | 220 |
| RT4 | Small irregular epithelial cells | I/II | T1 | 34 |
| J82 | Mixed epithelial and fibroblastic-like cells | III | T3 | 56 |
| TccSUP | Mixed epithelial and fibroblastic-like cells | IV+ | T4 | 34 |
| T24 | Mixed epithelial and fibroblastic-like cells | III | NR | <25 |
| HT1376 | Variable sized regular epithelial cells | III | T2/3 | <25 |
| TccDeS | Variable sized regular epithelial cells | II/III[a] | T4 | 3600 |

NR = not reported. Histology survey: Hepburn & Masters, 1983; Masters et al., 1986. [a]Institute of Pathology, The London Hospital.

Nevertheless, the fact that high levels were determined by an assay which is specific for the beta-chain, while negative results were obtained with a two-site assay specific for intact hCG, strongly suggests that the material consists principally of the beta subunit or fragments thereof. It is interesting that of the five ATCC (1985 catalogue) cell lines listed as hCG secretors, 4 are of placental/foetal origin (3 choriocarcinomas and one line of SV/40 transformed normal placental cells) and one is a cervical epidermoid carcinoma. The latter non-embryonal tumour cell line (CaSk1) is reported to secrete the beta subunit than intact hCG (Pattillo *et al.*, 1977). In a previous study, the hCG-like material found in urine and blood from patients with germ cell tumours appeared to be mainly intact hCG, while material from the patient with a metastatic bladder tumour consisted principally of free beta subunit (Norman *et al.*, 1985). Variability of the relative concentration of intact hormone and free subunits, together with heterogeneous glycosylation of the protein chains, has also been reported in studies on patients with a variety of other tumour types (Weintraub & Rosen, 1973; Rosen & Weintraub, 1974; Kahn *et al.*, 1977; Yoshimoto *et al.*, 1979*b*). The degree of glycosylation affects various biochemical characteristics and also the rate of metabolism *in vivo* (Van Hall *et al.*, 1971; Tsuruhara *et al.*, 1972). Independent production of subunit and intact hormone is not surprising as there is evidence that the genes for the alpha and beta subunits may be on different chromosomes. Thus, the alpha chain has been cloned (Fiddes & Goodman, 1979) and tentatively assigned to chromosome 6 (Naylor *et al.*, 1984), whereas the beta chain, also cloned (Fiddes *et al.*, 1980) has been mapped to chromosome 19 (Julier *et al.*, 1984). Furthermore, the beta hCG 'gene' is itself very heterogeneous consisting of a cluster of at least eight genes arranged in tandem and inverted pairs; one of this cluster is the gene for the beta chain of luteinising hormone (LH) (Boorstein *et al.*, 1982; Talmadge *et al.*, 1983).

The significance of this genetic complexity is unclear at the present time (Talmadge *et al.*, 1984; Whitfield & Kourides, 1985) but may partly explain the very variable results reported here and in the literature.

### References

ANDREWS, P.W., BRONSON, D.L., BENHAM, F., STRICKLAND, S. & KNOWLES, B.B. (1980). A comparative study of eight cell lines derived from human testicular teratocarcinoma. *Int. J. Cancer.*, **26**, 269.

ARMSTRONG, E.G., EHRLICH, P.H., BIRKEN, S. & 4 others (1984). Use of a highly sensitive and specific immunoradiometric assay for detection of human chorionic gonadotrophin in urine or normal, non-pregnant and pregnant individuals. *J. Clin. Endocrinol. Metab.*, **59**, 867.

BAYLIN, S.B. & MENDELSOHN, G. (1980). Ectopic (inappropriate) hormone production by tumors; mechanisms involved and the biological and clinical implications. *Endoc. Rev.*, **1**, 45.

BELLET, D., ARANG, J.M., CONTESSO, G., GAILLAND, J.M. & BOHUON, C. (1980). Localization of the beta-subunit of human chorionic gonadotrophin on various tumours. *Eur. J. Cancer*, **16**, 433.

BOORSTEIN, W.R., VAMVAKOPOULOS, N.S., FIDDES, J.C. (1982). Human chorionic gonadotrophin beta subunit is encoded by at least eight genes arranged in tandem and inverted pairs. *Nature*, **300**, 419.

BRAUNSTEIN, G.D., NAITUKAITIS, J.L., CARBONE, P.P. & ROSS, G.T. (1973). Ectopic production of human chorionic gonadotrophin by neoplasms. *Ann. Intern. Med.*, **78**, 39.

BRONSON, D.L., ANDREWS, P.W., VESSELLA, R.L., & FRALEY, E.E. (1983). In *Teratocarcinoma Stem Cells*, Silver, L.M. *et al.* (eds) **10**, p. 597 (Coldspring Harbour Conferences on Cell Proliferation) New York.

CHARD, T. (1978). The assay of alpha-fetoprotein. In *Prevention of Neural Tube Defects*, Crandall, B.F. and Brasier, A.D. (eds) p. 141. Academic Press: New York.

CHEN, H.C., HODGEN, G.D., MATSUWRA, S. & 6 others (1976). Evidence for a gonadotrophin from non-pregnant subjects that has physical, immunological and biological similarities to human chorionic gonadotrophin. *Proc. Natl Acad. Sci. USA*, **73**, 2885.

CIVANTOS, F. & RYWLIN, A.M. (1972). Carcinoma with trophoblastic differentiation and secretion of chorionic gonadotrophins. *Cancer*, **29**, 789.

COTTE, C.A., EASTY, G.C. & NEVILLE, A.M. (1981). Establishment and properties of human germ cell tumours in tissue culture. *Cancer Res.*, **41**, 1422.

DEXEUS, F., LIGOTHETIS, C., HOSSAN, E. & SAMUELS, M.L. (1986). Carcinoembryonic antigen and beta-human chorionic gonadotrophin as serum markers for advanced urothelial malignancies. *J. Urol.*, **136**, 403.

FIDDES, J.C. & GOODMAN, H.M. (1979). Isolation, cloning and sequence analysis of the cDNA for the alpha subunit of human chorionic gonadotrophin. *Nature*, **281**, 351.

FIDDES, J.C. & GOODMAN, H.M. (1980). The cDNA for the beta subunit of human chorionic gonadotrophin suggests evolution of a gene by readthrough into the 3′ untranslated region. *Nature*, **286**, 684.

HEPBURN, P.J. & MASTERS, J.R.W. (1983). In *The Pathology of Bladder Cancer*, Bryan, G.T. & Cohen, S.M. (eds) **2**, p. 213. CRC Press Inc.

HEYDERMAN, E., CHAPMAN, D.V., RICHARDSON, T.C., CALVERT, I & ROSEN, S.W. (1985). Human chorionic gonadotrophin and human placental lactogen in extragonadal tumours: An immunoperoxidase study of ten non-germ cell neoplasms. *Cancer*, **56**, 2674.

HUANG, S.-C., CHEN, H.-C., CHEN, R.-J., HSIEH, C.-Y., WEI, P.-Y. & OUYANG, P.-C. (1984). The secretion of human chorionic gonadotrophin-like substance in women employing contraceptive measures. *J. Clin. Endocrinol. Metab.*, **58**, 646.

JULIER, C., WEIL, D., COULLIN, P. & 7 others (1984). The beta chorionic gonadotrophin-beta luteinizing gene cluster maps to human chromosome 19. *Hum. Genet.*, **67**, 174.

KAHN, R.C., ROSEN, S.W., WEINTRAUB, S.D., FAJANS, S.S. & GORDEN, P. (1977). Ectopic production of chorionic gonadotrophin and its subunits by islet cell tumours. *New Eng. J. Med.*, **297**, 565.

KAWAMURA, J., MACHIDA, S., YOSHIDA, O., OSEKA, F., IMURA, H. & HATTORI, M. (1978). Bladder carcinoma associated with ectopic production of gonadotrophin. *Cancer*, **42**, 2773.

MASTERS, J.R.W., HEPBURN, P.J., WALKER, L. & 7 others (1986). Tissue culture model of transitional cell carcinoma: characterization of twenty-two human urothelial cell lines. *Cancer Res.*, **46**, 3630.

McILHINNEY, R.A.J. (1983). In *Current Problems in Germ Cell Differentiation*, McLaren, A. & Wylie, C.C. (eds) p. 175. Cambridge University Press.

McMANUS, L.M., NAUGHTON, M.A. & MARTINEZ-HERNANDEZ, A.M. (1976). Human chorionic gonadotrophin in human neoplastic cells. *Cancer Res.*, **36**, 3476.

NAYLOR, S.L., CHIN, W.W., GOODMAN, H.M., LALLEY, P.A., GRZESCHIK, K.H. & SAKAGUCHI, A.Y. (1983). Chromosome assignment of genes encoding the alpha and beta subunits of glyco-protein hormones in man and mouse. *Somatic Cell genet.*, **9**, 757.

NORMAN, R.J., LOWINGS, C., OLIVER, T. & CHARD, T. (1985). Human chorionic gonadotrophin and subunits – heterogeneity in serum of male patients with tumours of the genital tract. *Clin. Endocrinol.*, **23**, 25.

PATTILLO, R.A., HUSSA, R.O., STORY, M.T., RUCKERT, A.C.F., SHALABY, M.R. & MATTINGLY, R.F. (1977). Tumour antigen and human chorionic gonadotrophins in CaSK1 cells: a new epidermoid cervical cancer cell line. *Science*, **196**, 1456.

RODENBURG, C.J., NIEUWENHUYZEN KRUSEMAN, A.C., DEMAAKER, H.A., FIEUREN, E.J. & VAN OOSTEROM, A.T. (1985). Immunohistochemical localization and chromatographic characterization of human chorionic gonadotrophin in a bladder carcinoma. *Arch. Pathol. Lab. Med.*, **109**, 1046.

ROSEN, S.W. & WEINTRAUB, B.D. (1974). Ectopic production of the isolated alpha subunit of the glycoprotein hormones. *New Engl. J. Med.*, **289**, 1441.

ROSEN, S.W., WEINTRAUB, B.D. & AARONSON, S.A. (1980). Nonrandom ectopic protein production by malignant cells: direct evidence *in vitro*. *J. Clin. Endocrinol. Metab.*, **50**, 834.

SALEM, H.T., MENABAWEY, M., SEPPALA, M., SHAABAN, M.M. & CHARD, T. (1984). Human seminal plasma contains a wide range of trophoblast-'specific' proteins. *Placenta*, **5**, 413.

SEPPALA, M., IINO, K. & RUTANEN, E.-V. (1986). Placental proteins in oncology. *Clin. Obstet. Gynaecol.*, **14**, 593.

SHAH, U.M., NEWMAN, J., CROCKER, J. & 4 others (1986). Ectopic beta human chorionic gonadotrophin production by bladder urothelial neoplasia. *Arch. Pathol. Lab. Med.*, **110**, 107.

TALMADGE, K., BOORSTEIN, W.R. & FIDDES, J.C. (1983). The human genome contains seven genes for the beta subunits of chorionic gonadotrophin but only one gene for the beta subunit of luteinizing hormone. *DNA.*, **2**, 281.

TALMADGE, K., BOORSTEIN, W.R., VAMUAKOPOULOS, N.C., GETHING, M.J. & FIDDES, J.C. (1984). Three of the seven human chorionic gonadotrophin beta subunit genes can be expressed in the placenta. *Nucleic Acids Res.*, **12**, 8415.

TSURUHARA, T., DUFAU, M.L. & HICKMAN, J. (1972). Biological properties of hCG after removal of terminal sialic acid and glactose residues. *Endocrinology*, **91**, 296.

VAN HALL, E.V., VAITUKAITIS, J.L. & ROSS, G.T. (1971). Immunological and biological activity of hCG following progressive desialation. *Endocrinology*, **88**, 456.

VOLGELZANG, N.S., BRONSON, D.L., SAVINO, D. & FRALEY, E.E. (1983). In *Teratocarcinoma Stem Cells*, Silver, L.M. *et al.* (eds) **10**, p. 607 (Coldspring Harbour Conferences on Cell Proliferation) New York.

WEINTRAUB, B.D. & ROSEN, S.W. (1973). Ectopic production of the isolated beta subunit of human chorionic gonadotrophin. *J. Clin. Invest.*, **52**, 3135.

WHITFIELD, G.K. & KOURIDES, I.A. (1985). Expression of chorionic alpha and beta genes in normal and neoplastic human tissues: relationship to deoxyribonucleic acid structure. *Endocrinology*, **117**, 231.

YOSHIMOTO, Y., WOLFSEN, A.R. & ODELL, W.D. (1977). Human chorionic gonadotrophin-like substance in nonendocrine tissues of normal subjects. *Science*, **197**, 575.

YOSHIMOTO, Y., WOLFSEN, A.R., HIROSE, F. & ODELL, W.D. (1979*a*). Human chorionic gonadotrophin-like material: presence in normal tissues. *Amer. J. Obstet. Gynecol.*, **134**, 729.

YOSHIMOTO, Y., WOLFSEN, A.R. & ODELL, W.D. (1979*b*). Glycosylation: a variable in hCG production by cancers. *Am. J. Med.*, **67**, 414.